# Community health insurance amidst abolition of user fees in Uganda: the view from policy makers and health service managers

**DOI:** 10.1186/1472-6963-10-33

**Published:** 2010-02-04

**Authors:** Robert K Basaza, Bart Criel, Patrick Van der Stuyft

**Affiliations:** 1 Planning Department, Ministry of Health, Kampala, Uganda; 2 Public Health Department, Institute of Tropical Medicine, Antwerp, Belgium

## Abstract

**Background:**

This paper investigates knowledge of Community Health Insurance (CHI) and the perception of its relevance by key policy makers and health service managers in Uganda. Community Health Insurance schemes currently operate in the private-not-for-profit sector, in settings where church-based facilities function. They operate in a wider policy environment where user fees in the public sector have been abolished.

**Methods:**

Semi-structured interviews were conducted during the second half of 2007 with District Health Officers (DHOs) and senior staff of the Ministry of Health (MOH). The qualitative data collected were analyzed using the framework method, facilitated by EZ-Text software.

**Results:**

There is poor knowledge and understanding of CHI activities by staff of the MOH headquarters and DHOs. However, a comparison of responses reveals a relatively high level of awareness of CHI principles among DHOs compared to that of MOH staff. All the DHOs in the districts with schemes had a good understanding of CHI principles compared to DHOs in districts without schemes. Out-of-pocket expenditure remains an important feature of health care financing in Uganda despite blanket abolition of user fees in government facilities.

**Conclusion:**

CHI is perceived as a relevant policy option and potential source of funds for health care. It is also considered a means of raising the quality of health care in both public and private health units. To assess whether it is also feasible to introduce CHI in the public sector, there is an urgent need to investigate the willingness and readiness of stakeholders, in particular high level political authorities, to follow this new path. The current ambiguity and contradictions in the health financing policy of the Uganda MOH need to be addressed and clarified.

## Background

Community Health Insurance (CHI) schemes are voluntary arrangements, organized at the community level, that target people employed in the informal sector. They aim to improve people's financial access to health care. They run on a non-profit basis and apply the basic principle of risk sharing with community participation in design and management.

There is a growing interest in implementing CHI in the health systems of low and middle income countries for a number of reasons. First, many countries lack the capacity to levy sufficient tax revenue to finance a well-functioning health system. Insurance schemes offer an alternative channel to mobilize financial resources and increase access to health care. Second, CHI schemes can promote a client-oriented approach, ultimately empowering the "customer" [1]. Third, in order to meet the Millennium Development Goals (MDG), of which three relate to the health sector (reduction in child and maternal mortality rates and HIV prevalence), sustainable financial resources must be mobilized [2,3]. One of the recommended financing mechanisms in the African region is CHI [4]. The call for a more sustainable health financing mechanism such as CHI comes after the failure of the Bamako Initiative, which advocated for community financing of essential drugs and World Bank recipe of user fees. There was failure, however, to protect the poorest from the burden of payment and to let this group benefit preferentially and, ensure that their views were heard in decision-making [5].

There are studies on the functioning of particular CHI schemes but little is known about the perception and knowledge of managers of district health services and policy makers. Only two key studies in Africa have focused on knowledge and understanding of CHI by managers of health services. A study on the Maliando scheme in Guinea Conakry offered insight into perceptions of health providers at both operational and managerial levels [6], while another study looked at solidarity and financial sustainability based on an analysis of the values of CHI subscribers and promoters in Senegal [7].

### The context

Uganda is a low income country with a decentralized health system. Policy formulation and stewardship are the responsibility of the central government, while districts carry out implementation. A district is a self-governing administrative area; its health system is headed by a District Health Officer (DHO), who is both the technical and administrative manager of public health services and has a stewardship role of the entire district health system. The national health system consists of a public-owned health sub-system providing approximately 60% of health facilities, a Private Not for Profit (PNFP) sub-sector providing 30%, while the remaining (10%) of the facilities belong to the private for profit sub-sector. The total health care expenditure in Uganda is estimated to be US$20 per capita per annum, with 58% coming from out-of-pocket expenditure, 22% from the government and the remaining 20% from donors. Patients face difficulties in meeting hospital bills in places served by PNFP facilities, which are often the only providers in remote underserved areas. The health sector budget has been growing in real terms at a rate of 6%, while donor funding has remained constant since 2002. The health sector policy 1999/2000-2009/2010 provided for development of additional sustainable financial mechanisms provided that they did not adversely affect the poor. The Health Sector Strategic Plan 2004/5-2009/10 places community health insurance as a financing mechanism for the health sector. The strategic plan is a work program agreed to by all stakeholders. The ruling party's (the National Resistance Movement) presidential election manifesto (2006-2011) includes community health insurance schemes to improve delivery of health services. The manifesto considers CHI to provide protection against catastrophic health expenditure for both formal and informal sectors. Currently, the health sector is in the process of drafting a law to provide for CHI, and the initial process has been approved by the cabinet. Regulations and guidelines will be drawn up to conform to the law. Workshops for key policy workers and implementers have been arranged in the process of designing of the law and more will be carried out in the implementation process. Community health insurance is to be implemented in both the PNFP and public owned facilities. The fact that also government facilities are being targeted for CHI implementation is mystifying because user fees have been abolished in public health facilities, which theoretically makes CHI an irrelevant policy option. A possible explanation of this contradiction may be that, for a variety of reasons, the policy of user fee abolition is not effective and that, given the political sensitivity of the matter, implicitly, even insidiously, the way is opened to a decision where the abolition of user fee policy is abandoned and user fees are gradually being re-installed.

In an attempt to explore other health financing mechanisms, CHI schemes were first set up in 1996. These were started jointly by the Ministry of Health and donors, primarily the Department for International Development of UK (DFID) and United States Aid for International Development (USAID). All the existing schemes were based on or linked to PNFP health facilities. An inventory of the Ugandan CHI schemes done in 2007 by the Uganda Community Based Health Financing Association (UCBHFA) indicates that there are fourteen schemes. Total membership was 100,000 people with varying coverage from 5-10% of the catchment population and contributing 5-10% of the facility budgets. Schemes were implemented in faith-based hospitals because they still charge user fees, are quite widely used and generally perceived as providing good quality of care. The UCBHFA annual report of 2007 points out that there has been additional quality improvement to meet demands by members of the schemes; examples are a reduction in waiting times, introduction of laboratory services and availability of qualified staff to treat scheme members. Schemes exist in only 9 out of 82 districts in the southern part of the country. Most of the schemes cover both in-patient and out-patient care, and the premium is on average US$5-10.00 per person per year. In all the schemes, members pay a small co-payment at the time of service.

The government in 2001 abolished user fees in the general wings of public-owned hospitals and lower level health facilities, but fees remain in private wings of government hospitals and in all private facilities. There are doubts about whether funding of public facilities is adequate to ensure that the population can access quality care without having to pay fees at the point and time of utilization. Country medicines surveillance data points to a key concern: medicines are frequently not available. Only 28% of the sampled health facilities surveyed in 2007 had continuous availability of the six tracer medicines (cotrimoxazole, oral rehydration salts, medroxyprogesterone, sulfadoxine pyrimethamine, measles vaccine and coartem) [8]. For this and other reasons, many Ugandans still have to make out-of-pocket payments to secure adequate health care or at least purchase medicines in private sector health facilities. In the PNFP facilities, user fees provide over 50% of hospital income. It is in this particular policy context that CHI options are thus considered and our investigation was carried out. The number of schemes and the persons covered remain small and are confined to one part of the country, despite CHI being earmarked as a preferential health financing mechanism in the current health policy and the health sector strategic plan [9]. Moreover, an exploratory study done in 2005 hypothesized that the lack of a coherent policy framework to develop CHI, amidst a backdrop of user fee abolition in the public sector, and the lack of information and understanding of CHI by district services managers and central staff in the Ministry Of Health (MOH), are contributing factors to low enrollment in CHI schemes [10]. The current study is an in-depth investigation into the knowledge and understanding of health financing policies in general and policies on CHI in particular by policy makers and key implementers.

### The objective of the study

The objective of the present study is to determine the level of knowledge and understanding of CHI and the perception of its relevance among key policy makers and district health service managers in Uganda. More specifically, three research questions were formulated:

(1) Do DHOs and senior MOH staff understand the basic principles of CHI? (2) Do DHOs and senior MOH staff know the status of existing CHI schemes in Uganda? (3) What is the perception among these people of the relevance of CHI amidst a national policy of abolition of user fees in the public sector?

## Methods

This qualitative study was conducted during the second half of 2007 and involved two study populations: District Health Officers and senior staff at the Ministry of Health headquarters. Of 64 senior MOH staff, 29 were interviewed. Purposeful sampling was used in selecting staff from among the five major departments at the Ministry of Health headquarters: Finance and Administration, National Disease Control, Quality Assurance, Clinical Services and Community Health. The staff in the Planning Department, where the promotion of CHI schemes is based, was excluded; they had previously been interviewed in a related study [11]. DHOs in districts with schemes and without were interviewed. Simple random sampling of 43 DHOs from a sample frame of 73 districts without CHI schemes was carried out. The nine DHOs in districts with existing schemes were later interviewed. The results of the interview were used to verify responses, provide additional information and for comparison. These individual participant interviews were semi-structured and administered using a topic guide (Appendix 1). Telephone interviews were used for DHOs because they are flexible, relatively reliable and cost less to conduct. We used face-to-face interviews, although less efficient, at MOH headquarters because they are reliable, more convenient. In addition, the staff of MOH headquarters were located in Kampala, the base of the study. Pilot testing was done on two staff at MOH headquarters and two DHOs from districts without schemes who were not part of the sample for the study. All the interviewees were contacted at least a week in advance and appointments were set for the interviews. Two university researchers were recruited to carry out the interviews and were trained for one day. All interviews were carried out in English. The total number of cadres interviewed was 81 and interviews lasted 30 minutes on average. Transcription of interviews was done verbatim on the same day and scripts were double-checked. The "framework" method was used for data analysis [12]. Indexing and analysis were completed along the lines of the three aforementioned research questions. EZ-Text software facilitated tabulation of frequencies of indexed transcripts.

For ethical considerations, the researchers explained to the interviewees that results of this study will remain anonymous and be used only to contribute to the ongoing policy development of CHI in the health sector; explicit verbal consent was obtained. Permission was also sought from the interviewees to take notes and to audiotape the interviews. This study is part of the planned work program on financing of the health sector.

## Results

The results of the interviews were consolidated and the responses quantified. Figures given in brackets indicate the number of quotes that were collected pertaining to the specific issue. For example, the index (DHO1) indicates that one quote with the specific issue of concern was collected from a District Health Officer and (DHO 2) denotes two quotations from DHOs. The same applies to interviews from staff of Ministry of Health Headquarters. Plain numbers between brackets, for example (10), indicate that the issue was directly mentioned ten times in the interviews. Similarly, (V1) is used to indicate one quote regarding an issue of concern collected from a District Health Officer with a scheme. Ellipses are used to denote missing speech. Quantification of direct responses has been used to contribute to full understanding of respondent's opinions.

The majority of the respondents were middle aged, male and with at least post-bachelor's degree training and more than 10 years of working experience in the health sector (Table 1).

**Table 1:** Respondent characteristics of DHOs in districts without schemes and staff of MOH headquarters

		DHO(n = 43)	MOH(n = 29)
Age	Less than 30 years	--	1(4%)
	30-50 years	39 (91%)	20 (69%)
	Above 50 years	4 (9%)	8 (27%)
Sex	Male	41(95%)	19 (68%)
	Female	2 (5%)	9 (32%)
Educational level	Below a Bachelors degree (diploma)	--	4 (14%)
	Bachelors degree	-	4 (14%)
	Post graduate degree qualification	43 (100%)	21 (72%)
Work experience in the health sector	Less than 10 years	--	1(4%)
	More than 10 years	43 (100 %)	28 (96%)

### General knowledge and understanding of CHI

In total, 95% of DHOs in the districts without schemes and 90% of the MOH staff said they "had heard" about the concept of CHI. The most common sources of information were direct contact with fellow health workers (DHO 10 and MOH 12), media (DHO 16 and MOH 4), workshops and seminars (DHO 16 and MOH 2) and during university studies (DHO 14 and MOH 2), Table 2. DHOs with schemes (V) said their sources of information were reports from the schemes (V5), workshops and seminars (V3) and study at university (V3), Table 3.

**Table 2:** Sources of information by DHOs in districts without schemes and staff of MOH headquarters

	DHO	MOH
1. From direct contact with other health workers	10 (16%)	12 (55%)
2. Media (news-papers, radio and TV)	16 (26%)	4 (18%)
3. Workshops and seminars	16 (26%)	2 (9%)
4. At university	14 (22%)	2 (9%)
5. Visit to communities with schemes	6 (10%)	2 (9%)
Total number of responses	63 (100%)	22 (100%)

**Table 3:** Sources of information by DHOs in districts with schemes

	V
1. From direct contact with other health workers	5 (48%)
2. Workshops and seminars	3 (26%)
3. At university	3 (26%)
Total number of responses	11 (100%)

DHOs and staff of the MOH who indicated they had heard of CHI were asked to explain its main principles. In addition, probing was done using five important characteristics of the CHI concept (i.e., pooling of pre-paid funds, a dynamic of mutual aid, targeting of the informal sector, not-for-profit characteristics and community participation in management). Two-thirds of the MOH staff and a fifth of DHO interviewed could not name more than two characteristics after probing.

However, some DHOs and MOH staff had a good understanding of the principles of CHI:

"It is an organized system for the sake of getting medical care, where a fee is subscribed per year and one is covered when one is sick; if one is not sick it pays for someone else who is sick." (DHO)

"It is a guarantee for services; if you have contributed together, family members can access services. It is not for profit and the community participates in its leadership." (MOH)

The DHOs in the districts that had schemes each cited at least three characteristics of CHI schemes.

We also investigated interviewees' perceived strengths of CHI. Common strengths mentioned were removing the fear of inability to pay when sick (36), improving quality of health services (20) and raising financial resources for health care (9). The respondents were asked what they perceived to be the limitations of CHI. The most cited were the possibility of funds being mismanaged (26), difficulty in mobilizing the informal sector workers to become members of the scheme (18) and problems in people's ability to pay (16).

The DHOs with schemes stated that the strengths of CHI were that it increases access and early seeking behavior because patients do not have to first look for cash when sick (5), empowers the community to demand better quality care (2) and enables the facility to plan for better services, such as buying drugs in advance (1).

"The advantage is that you get services, even when you do not have money at the moment of sickness, because you will have pre-paid." (V1)

These DHOs mentioned limitations such as the risk of mismanagement of funds (DHO 4), failure to afford the plan (DHO 3), current poor quality of services (e.g., shortages of drugs and health workers) (DHO 2), and that people may not fall sick and yet have pre-paid for services (DHO 1).

"The limitation is for people who do not fall sick; for example, my family and I can take a whole year without falling sick and I may feel that I am not benefiting." (DHO 1)

### Knowledge on existing situation of CHI schemes in Uganda

The interviewees who had heard of the CHI concept were also asked whether they were aware that CHI schemes had been operating in Uganda for some years. Over half (62%) of the MOH staff interviewed and close to a half of the DHOs in districts without schemes (42%) were not aware of this. The same interviewees were asked if they knew that CHI is only implemented in PNFP health facilities; 60% of MOH staff and close to a half of the DHOs in districts without schemes were unaware of it. The primary reasons for implementation of CHI in PNFP and not in public-owned facilities that interviewees gave were: people have confidence in health providers/good quality of care (DHO 4). Other reasons are existing user fees in PNFP facilities (MOH 5 and DHO 2), and trusted and better managers in PNFP sub-sector (MOH 5 and DHO 2). Free services in public-owned units (DHO 9 and MOH 4) were mentioned as a reason for the absence of CHI schemes.

"In the PNFPs, there are user fees unlike in government facilities, in which people think that everything is free. It would be hard to implement CHI in public-owned facilities when the government has pronounced itself that there are no user fees. You see, it would be a surprise." (DHO)

The DHOs in districts with CHI schemes pointed out that these exist in PNFP facilities because of the presence of user fees (V6), and because services are perceived to be of better quality in terms of availability of medicines and health workers (V3) compared to publicly-owned facilities.

"It is appropriate to start the schemes where there are user fees and thus pre-payment is relevant, but this is not applicable in government units because services are presumed to be free." (V1)

Of the interviewees knowledgeable about CHI, 72% of the DHOs and 45% of the MOH staff were unaware of the existence of the umbrella organization for CHI schemes: the Uganda Community Based Health Financing Association. Among DHOs in districts with schemes, seven out of nine (80%) had ever heard of this association. None of the DHOs in either category of district or MOH staff had used the services of this association. In regard to CHI being part of the sector strategic plan, slightly less than a quarter (24%) of the staff of Ministry of Health headquarters and 90% of the DHOs in districts without schemes were aware of this, while all the DHOs with schemes were aware. The interviewees were asked about their perceptions of the expectations of Ugandan policy makers vis-a-vis CHI. The most common answers were that CHI may lead to improved health services, including better quality, availability of medicines and accessibility (DHO 11 and MOH 10). Also, CHI may raise money for health services (DHO 14 and MOH 2). DHOs in districts with schemes had expectations that CHI may contribute to financing of health services (V2) and raise quality of health services (V2). It was expressed that before expectations are met, intense sensitization would be needed (DHO 3 and MOH 3).

"Sensitization of all stakeholders should be carried out, starting with policy makers like the parliament and district councils, health workers and communities." (V1)

### Relevance of CHI in the framework of abolition of user fees in public-owned facilities

The interviewees who had heard of CHI (DHO N = 41 and MOH N = 24) were asked to express an opinion on the relevance of CHI amidst a public policy of abolition of user fees.

Over half of DHOs and over a third of MOH staff interviewed perceived CHI as being a relevant policy option. CHI was mainly seen as a way of improving quality of care and as a means of raising additional money for health care (Table 4 and 5).

**Table 4:** Opinions on relevance of CHI amidst abolition of user fees by DHOs in districts without schemes and by MOH headquarters staff

	**D**HO	MOH
1. Is a way of improving quality and access to care	2 (28%)	11 (74%)
2. Raising additional money for health care	16 (37%)	--
3. Money is prepaid and pooled	13 (30%)	2 (13%)
4. Better way of planning/managing health services	2 (5%)	2 (13%)
Total number of responses	43 (100%)	15 (100%)

**Table 5:** Opinions on relevance of CHI amidst abolition of user fees by DHOs in districts with schemes

	V
1. As way of improving quality of services and access to care	8 (89 %)
2. In government units only when there are user fees	1 (11 %)
Total number of responses	9 (100%)

"Recently, user fees were abolished but there are shortages of medicines and other health supplies. It turns out that one has to pull money from his pocket. As much as we think that services are free, there are out-of-pocket expenses. I think what would make CHI relevant is that it would be something official." (DHO)

However, some respondents from the Ministry doubted the relevance of CHI:

If comparing the two, I would prefer user fees; I go to the hospital ... aware that if I have money, I will get treatment. But with CHI, I don't know whether there is medicine, and even if it is there, the health worker may tell you that it's not there. With user fees, they will give you the medicine because you are going to pay for it." (MOH)

When asked if patients faced out-of-pocket health expenditures in general wings of public-owned hospitals and health centers, all DHOs and MOH headquarters staff interviewed affirmed that patients make out-of-pocket payments when using public health services. According to the interviewees, most of the out-of-pocket expenditure is used for buying medicines and other health supplies from private sector outlets like clinics, pharmacies, drug shops, and for informal payments to health workers (Table 6 and 7).

**Table 6:** Explanations for out-of-pocket expenses for health care by DHO in districts without schemes and by MOH headquarters staff

	DHO	MOH
1. Buy drugs and other medical supplies	30 (68%)	13 (59%)
2. Informal payments	13 (30%)	5 (23%)
3. Buying stationary for writing patient's notes	1 (2%)	2 (9%)
4. Paying for laboratory services	--	2 (9%)
Total number of responses	44 (100%)	22 (100%)

**Table 7:** Explanations for out-of-pocket expenses for health care by DHO in districts with schemes

	V
1. Buy drugs and other medical supplies	4 (44%)
2. Informal payments	3 (33%)
3. Buying stationary for writing patient's notes	1 (23%)
Total number of responses	8 (100%)

Patients face out-of-pocket expenses, even in a big government hospital (name withheld) you have to buy gloves in the labor ward; if you do not pay you suffer." (MOH)

"Some people are being charged unofficially. If CHI is in place, it is likely to replace these unofficial charges and in the long run ... may improve the health services." (DHO)

".....the government does not provide enough funds to buy medicines; patients are referred to outside government-owned units to buy medicines." (V1)

Interviewees were also asked about the future role of CHI in Uganda. All the DHOs of districts with and without CHI schemes, and over half (55%) of the staff of MOH perceived CHI to have a future in the country.

"Because there is community contribution, there will be motivation of health workers and increased funding for the health sector." (DHO)

"There is a bright future for CHI scheme. People will subscribe, including me." (MOH)

"Health is something that everyone needs to maintain, and therefore CHI has a place in Uganda. Let us start with national policies facilitating CHI...Regulations are very important and gradual implementation is needed." (MOH)

"CHI should have been established a long time ago; somehow the community should make contributions. People should know that there are no free things, because what is perceived to be free, after all, is not free. Secondly, the community needs ownership of such schemes."(V1)

However, there were also some dissenting views on CHI:

"CHI presents a conflict of policy with abolition of user fees." (MOH)

"There is need of a mandate on how things should function, and apparently there is no policy in place." (MOH)

"The schemes are a duplication of services because government is providing free drugs to all health centers at the moment. What is the use of these schemes? It would bother the community because people are poor and do not have money." (MOH)

"No, I do not think that the scheme can run hand in hand with abolition of user fees; they cannot go together. They are mutually exclusive and, of course, for the scheme to succeed user fees must be in place." (DHO)

## Discussion

There was poor knowledge and understanding of the principles and activities of CHI in Uganda by staff at MOH headquarters and by DHOs in districts without schemes. Community health insurance is a relatively new subject in Uganda and therefore the research called for a clear list of issues to be asked about during the interviews. Most of the health services are located in urban areas and offer poor quality services, whereas the majority of CHI members live in the rural areas. This affects enrollment into schemes. There is lack of understanding of the principles of insurance, such as the expectation of benefit even if not ill. The primary benefit of CHI is to provide access to health care when one is sick and avoid concern about costs of treatment. There has never been any specific national conference, guidelines or deliberate attempt by the MOH to promote CHI in public units. This may explain the low level of knowledge of CHI.

In the study of perceptions of health providers at both operational and managerial level in the Maliando CHI scheme in Guinea Conakry, lack of understanding by health managers was a contributing factor to low scheme enrollment [6]. In another study, conducted in Senegal, it was found that implementation of CHI schemes has been slow and laborious. This was explained in part by tension between the competing objectives pursued by both promoters and subscribers [7]. In addition to minimal knowledge and understanding of CHI by providers and policy implementers, studies have indicated that poor knowledge and understanding on the part of the schemes beneficiaries is also a contributing factor to low enrollment in Uganda [10,13].

The central level staff may have been expected to know more about CHI principles and other strategic plan issues compared to periphery staff, but we found the opposite: 90% of the DHO in districts without schemes compared to 24% of the MOH were aware of CHI. The possible explanations are that DHOs are more often confronted with the operational implications of health financing than MOH headquarter staff. The majority of MOH staff work on vertical programs and may not be familiar with the health system issues. The DHOs, by virtue of their position, are explicitly involved in the drafting and dissemination of the health sector strategic plan in their districts. Furthermore, the DHOs develop district strategic plans that often have some semblance to the national plan. The explanation for a larger proportion of the MOH staff (55%) aware of the existence of Uganda Community Based Health Financing Association (UCBHFA), compared with 28% of the DHOs in districts without schemes may be due to UCBHFA being a central institution that is more likely to interact with MOH headquarters than DHOs in districts without schemes. Two thirds of the DHOs in districts with schemes are aware of the existence of UCBHFA. However, they may have received additional information from existing schemes in their districts. This analysis points to gaps and differences in knowledge and understanding of CHI between central level MOH staff and the periphery managers (DHOs) that should be addressed by the Uganda health sector if it moves CHI higher on the health care agenda.

The results of our study suggest that seminars, the use of media and visits to communities with schemes are sources of information on CHI. If the health sector in Uganda was to consider CHI, it will be necessary to develop a sensitization and information strategy on CHI to properly inform all stakeholders. The sector could consider using the aforementioned channels for information. The DHOs with schemes in their districts pointed out that if CHI was introduced as a replacement for abolition of user fees, payment for services may be more transparent and thus, a possible substitute for under-the-table payments. Once members have made official payments and organize a scheme, they are likely to resist under table payments. Moreover, they can report health workers involved in the practice of taking under-the-table payments. CHI has the potential of modifying the power relationship of providers and members of the scheme in favor of the latter [4]. The money collected in the schemes could also be used to buy supplementary medicines and other health supplies.

Out-of-pocket expenditure remains the major health care financing mechanism in Uganda despite abolition of user fees in government facilities. In the study on abolition of user fees in Uganda by the World Health Organization country office [11], there was no deterioration of medicine stock reported as a result of the abolition of user fees. Seven years later the findings are different; interviewees think that basic medicines and other medical supplies are lacking in public facilities, quality of care is poor, and there is a generalized practice of under-the-table payments. This may indicate that the policy of abolition of user fees may not have led to the desired improvements in health care delivery. The results of our study are important in light of the findings in two related experimental field studies in Cameroon and Niger, in which user fees accompanied by quality improvements increased equity and access to health care, especially for women, children and the poor [14,15]. The results of these two studies, however, must be interpreted against a background that they were small pilots. They cannot be generalized on a nationwide basis and we do not know whether they can be sustained over time.

This study indicates that CHI is perceived as being a relevant policy option for Uganda; more specifically it is seen as a potential source of funds and as a means of raising the quality of care. Respondents may feel that the quality of care will be improved due to increased availability of health workers and medicines. There is moderate evidence in the literature to suggest that community-based health insurance schemes may have a positive effect on resource mobilization in the areas where they operate. There is, however, weak or no evidence that CHI schemes have an effect on the quality of care or the efficiency with which care is produced [16,17].

Despite assurances provided at the start of the interviews, some of the interviewees may not have felt free to express their opinion on a sensitive issue such as abolition of user fees. One of the MOH headquarters staff and two DHO actually refused to be audio-taped. DHOs may not have been interviewed previously on telephone; indeed one DHO from a district with schemes insisted on traveling to Kampala, the base of study, for a face-to-face interview. Community health insurance is sometimes a controversial and politically sensitive issue in Uganda, where user fees have been abolished in the public sector following a decision by the president. We acknowledge these two study limitations.

## Conclusion

We trust that our results will inform the ongoing process of CHI development in Uganda, as well as policy makers and implementers in similar settings that have CHI on their agenda. As far as the PNFP sub-sector is concerned, user fees will probably continue to be charged unless subsidies dramatically increase. Until then, CHI remains a relevant option for the PNFP sub-sector. Out-of-pocket payments at the point and time of health service utilization in order to meet severe shortages of supplies seem to be a generalized and increasing practice in the public sector in Uganda, despite the official policy of abolition of user fees. This study has pointed out clearly the ambiguous government policies in the field of health care financing. The Ugandan health sector will have to make explicit choices and investigate whether and why the abolition of user fees in the public sub-sector has produced or not the desired results; only then can a clear evidence-based policy be established with regard to the possible introduction of CHI in the public sub-sector.

What are the policy options for the Ministry of Health? One is to keep CHI implementation restricted to the PNFP sector, as is the case today. The public funding of the government health sector would then need to be dramatically increased so as to make the abolition of user fees at facility level (again) a reality. If, however, the Uganda health sector intends to go ahead with CHI in the public sector, then it implicitly means that the decision is taken to abandon the policy of abolition of user fees and to reintroduce user charges in the public sector; otherwise CHI would be an irrelevant policy option. In this case, clear and unambiguous communication is required from the MOH. A third and intermediate strategy would be a selective introduction of CHI in the public sector for some services. Under that scenario, CHI would fill the gaps in local health care delivery and in the current social health protection coverage. Pooled prepayments, instead of the current individual out-of pocket payments at the time of use that are apparently taking place on the ground, would then be organized for services and activities perceived by people as a priority.

Whatever option taken, it is clear that CHI on its own is not and will not be the ultimate solution to health financing problems in the Ugandan sector. CHI should be considered among other health financing reforms like increased budget allocation in line with Abuja declaration (15% of total public funds going to the health sector) and mobilization of external resources like donations, grants and loans. A comprehensive and unambiguous health financing strategy that addresses all avenues of resource mobilization including additional government funding in line with Abuja declaration is important. Efficiency and equity must be central in this strategy.

The health system should, in as much as possible, reduce the occurrence of out-of-pocket payments that constitute a barrier to the utilization of health care. In that respect, CHI is theoretically a relevant strategic option. However, in order to fully explore whether it is feasible to introduce CHI in Uganda in the current context, there is urgent need to investigate the willingness of the primary stakeholders, in particular political authorities at the highest level, to follow this new path. In terms of ownership and vision, both politicians and Ministry of Health technicians may have to be involved in charting this course.

## Competing interests

The authors declare that they have no competing interests.

## Authors' contributions

RB designed, carried out the study, produced the first draft and revised the manuscript. BC and PVDS designed the study and revised the manuscript. All authors have read and approved the submission of the manuscript to BMC Health Services Research in its present form.

## **Appendix 1** Topic guide

1. Have you ever heard about the concept of Community Health Insurance (CHI), or Community-Based Health Insurance (CBHI)? If yes, go to the next question. If no, stop interview.

2. If yes, where and how did you first hear about it (at university, at the MOH, at a workshop, in policy documents, elsewhere)? Please explain.

3. Are you aware of the fact that in some parts of Uganda CHI schemes have been operating for some years now? If yes, how did you come across this knowledge? And could you name one or more places where this new strategy is being implemented?

4. Currently in Uganda, CHI is only implemented to cover user fees in private not-for-profit health care facilities, like faith-based or church-based hospitals. Did you know that? Why do you think this is the case? Please elaborate.

5. Could you briefly explain what the basic principles of CHI are? Please take your time to explain to me how you understand CHI.

The interviewers probe respondent's answer(s) on:

*Sharing of the financial cost of health care via pooling of pre-paid funds

*Dynamic of mutual aid

*Targeting informal sector

*Not-for-profit character

*Some degree of community participation in design and management

*Other...

6. According to you, what are the primary strengths (or advantages, or potential) of CHI? Please elaborate. What would be the primary weaknesses (disadvantages, limitations) of CHI? Please elaborate.

7. Currently in Uganda, no fees are paid for utilization in the government health facilities (see policy of abolition of user fees in public facilities). In that context, what is your opinion on the relevance of CHI? Do you believe CHI to be a worthwhile option for both public and PNFP sub-sectors? If yes, please explain why? If not, please explain why not?

8. Are you aware that CHI is part of the second Health Sector Strategic Plan II (2005/6-2009/10) of the Ministry of Health? If yes, can you say something about the expectations policy makers have vis-à-vis CHI? Please elaborate.

9. Have you ever heard about the Uganda Community Based Health Financing Association (UCBHFA)? If no, go to the next question.

- If yes, how did you come across that association?

- What purpose does this association serve?

10. Have you ever used its services? As mentioned above, currently in Uganda no user fees are charged in government facilities. In your opinion, does this mean that the patients who use government facilities do not face an out-of-pocket expenditure? Please elaborate.

- If indeed, in your opinion, there is some level of out-of-pocket expenditure by patients using government facilities, would there then be a place for CHI? Please elaborate.

11. Currently, the enrollment rates in the dozen existing CHI schemes in Uganda remain very low. What would be in your opinion the primary reasons for that situation? Why would people be reluctant to enroll? What are the primary obstacles? Please elaborate.

12. Having said all this, do you believe there should be a place now or in the future for CHI in Uganda? Please elaborate.

## Pre-publication history

The pre-publication history for this paper can be accessed here:

http://www.biomedcentral.com/1472-6963/10/33/prepub
